# Evaluation of Multiparametric Magnetic Resonance Imaging in Detection and Prediction of Prostate Cancer

**DOI:** 10.1371/journal.pone.0130207

**Published:** 2015-06-12

**Authors:** Rui Wang, He Wang, Chenglin Zhao, Juan Hu, Yuanyuan Jiang, Yanjun Tong, Ting Liu, Rong Huang, Xiaoying Wang

**Affiliations:** 1 Department of Radiology, Peking University First Hospital, Beijing, China; 2 Department of Radiology, First Affiliated Hospital of Kunming Medical University, YunNan, China; 3 Department of Radiology, Aerospace Central Hospital, Beijing, China; 4 Department of Radiology, Dongzhimen Hospital, Beijing, China; 5 Department of Radiology, Peking University Shenzhen Hospital, Guangdong, China; Chinese Academy of Sciences, CHINA

## Abstract

**Background:**

Although European Society of Urogenital Radiology proposed the potential of multiparametric magnetic resonance imaging (MP-MRI) as a tool in the diagnostic pathway for prostate cancer (PCa) and published a unified scoring system named Prostate Imaging Reporting and Data System (PI-RADS version 1), these still need to be validated by real-life studies.

**Objective:**

To evaluate the role of MP-MRI in detection and prediction of PCa.

**Methods:**

Patients with clinical suspicion of PCa who underwent prebiopsy MP-MRI from 2002 to 2009 were recruited. MP-MRI results were retrospectively assigned as overall scores using PI-RADS by two radiologists. Patients were followed and the end point was the diagnosis of PCa. Receiver operating characteristics (ROC) curve was performed to test diagnostic efficacy of MP-MRI, under results of biopsy within three months. The cox proportional hazards model was used to identify independent variables for the detection of PCa.

**Results:**

Finally, 1113 of the 1806 enrolled patients were included for analysis. The median follow-up was 56.0 months (1–137 mo). For 582 patients biopsied within three months, area under the curve for the detection of PCa with MP-MRI was 0.88 (95% confidence interval [CI], 0.75–1.00) in group of baseline prostate specific antigen (PSA) 0.01–4.00 ng/ml (n = 31), 0.90 (95% CI, 0.84–0.95) in PSA 4.01–10.00 ng/ml (n = 142), and 0.91 (95% CI, 0.87–0.94) in PSA >10.00 ng/ml (n = 409), respectively. In the cox model adjusted for age and baseline PSA level, for the detection rate of PCa, compared with PI-RADS 1–2 (reference), the hazard ratio was 6.43 (95% CI, 4.29–9.65) for PI-RADS 3, 18.58 (95% CI, 13.36–25.84) for PI-RADS 4–5 (p < 0.001).

**Conclusions:**

Prebiopsy MP-MRI with PI-RADS is demonstrated as a valuable diagnostic and predictive tool for PCa.

## Introduction

It has been shown that multiparametric magnetic resonance imaging (MP-MRI) is useful for the detection of prostate cancer (PCa) [1,2]. However, the current view is that magnetic resonance imaging (MRI) still has a limited role in the clinical management of PCa [3,4]. The worldwide acceptance of the role of MP-MRI was hampered by the variations in MRI protocols, absence of unified diagnostic criteria and few validation prospective studies [3,5–7].

To solve the problems, European urology and radiology proposed a unified scoring system for MP-MRI in 2012, named Prostate Imaging Reporting and Data System (PI-RADS), to standardize the reporting system, promote the diagnostic value of MP-MRI, and facilitate communication between clinicians and radiologists [8]. Meanwhile, European Society of Urogenital Radiology (ESUR) experts drew a conclusion that MP-MRI, with a host of reports manifesting its potential as a diagnostic tool for PCa, was going through a period of development [9].

Nevertheless, this opinion drew a quick response of editorial that MP-MRI was still not ready for routine use [10]. Meanwhile, PI-RADS, based on literature evidence and consensus expert opinion from ESUR, still lack validation in a real-life setting. So, our study was to evaluate the role of MP-MRI with PI-RADS in detection and prediction of PCa in Chinese patient population.

## Materials and Methods

### Patient selection

**Fig 1:**
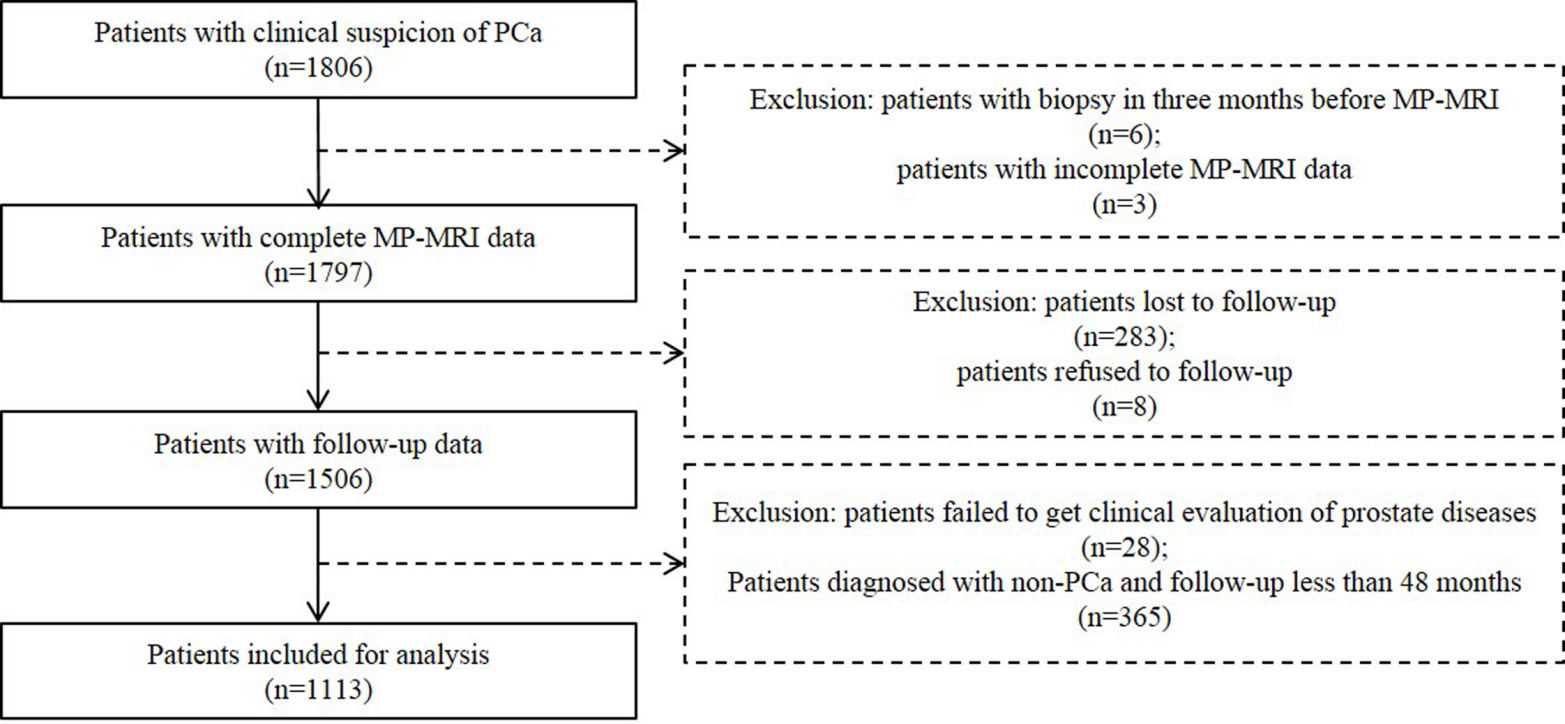
Inclusion and Exclusion Criteria for Patients Included in Data Analysis. PCa: prostate cancer; MP-MRI: multiparametric magnetic resonance imaging.

The study was approved by the institution ethics committee of Peking University First Hospital. Between July 2002 and December 2009, 1806 consecutive patients with clinical suspicion of PCa (elevated prostate specific antigen (PSA), abnormal digital rectal examination (DRE) or family history of PCa), underwent prostate MRI prior to biopsy, were recruited. All the patients provided written informed consent. Before prostate MRI, a questionnaire survey, including the latest PSA level within one month, DRE, previous biopsies, previous prostate MRI scans, history of previous prostate treatment or intervention, was conducted. Patients were consistently followed with intervals of one to two years and were censored at the occurrence of the end point of PCa, emigration, or 31 September 2014, whichever came first. At the end of follow-up period, final diagnosis was made according to pathology (biopsy, transurethral resection of the prostate (TURP) or surgical pathology) or clinical comprehensive analysis based on long-term follow-up by PSA, DRE, transrectal ultrasound (TRUS) or MRI. The following data were excluded from analysis: (a) patients with previous biopsy in three months before prostate MP-MRI; (b) patients with incomplete MRI data; (c) patients lost or refused to follow-up; (d) patients failed to get clinical evaluation of prostate diseases because of other severe comorbid conditions; (e) patients with diagnosis of non-PCa and follow-up time less than 48 months. After the exclusion of 693 patients for various reasons, 1113 patients were included for analysis (Fig 1).

### MRI Protocol

All scans were obtained with a 1.5 T MR scanner with the integrated endorectal-pelvic phased-array coil (GE Medical System, Milwaukee, Wis). MP-MRI was done including a combination of high-resolution T2-weighted imaging (T2WI) and at least two functional MRI techniques (diffusion-weighted imaging (DWI), magnetic resonance spectroscopy (MRS) and (or) dynamic contrast enhanced imaging (DCEI)). The MP-MRI parameters are presented in Table 1.

**Table 1:** MP-MRI Parameters at 1.5 T.

	1.5 T MR*			
	T2WI	DWI	DCEI**	MRS***
Repetition time (msec)	3500	3500	4	1000
Echo time (msec)	85	56.4	1.9	130
Field of view (mm^2^)	240×240	260×260	360×360	110×110
Acquisition matrix	320×256	128×128	256×256	16×8×8
Flip angle (degrees)	90, 180	90	15	180
b Values (sec/mm^2^)	…	0, 800	…	…
Section thickness (mm)	4	5	3.8	…
No. of temporal acquisitions	…	…	15	…

MP-MRI: multiparametric magnetic resonance imaging; MR: magnetic resonance; T2WI: T2-weighted imaging; DWI: diffusion-weighted imaging; DCEI: dynamic contrast enhanced imaging; MRS: magnetic resonance spectroscopy.

* Signa Twinspeed; GE Medical System, Milwaukee, Wis. Used with a 8-channel pelvic phased array coil and an endorectal coil.

** An intravenous injection of 0.1 mmol/kg of gadopentetic acid dimeglumine salt injection (Bayer Schering Pharma, Germany) was performed at 2.0 ml/sec, with saline flush of 15ml.

*** Three-dimensional MRS was performed using the Point Resolved Selective Spectroscopy sequence.

### Reporting Protocol

The prostate MR images were retrospectively interpreted by two radiologists with 3 years (RW) and 10 years (HW) of experience. Readers were not blinded to the questionnaire survey written by each patient before MRI. According to PI-RADS version 1 [8], a five point subjective suspicion overall score was assigned to all focal abnormalities: score 1: clinically significant disease is highly unlikely to be present; score 2: clinically significant cancer is unlikely to be present; score 3: clinically significant cancer is equivocal; score 4: clinically significant cancer is likely to be present; score 5: clinically significant cancer is highly likely to be present.

### Reference Standard

Prostate biopsy was performed under the guidance of TRUS. To calculate the diagnostic efficacy of MP-MRI, score 1–2 of PI-RADS were considered negative findings and score 3–5 were considered positive findings. Biopsy within three months after MP-MRI was defined as the reference standard.

### Patients with Negative Initial Biopsy and Final Diagnosis of PCa

Patients with negative initial biopsy results and final diagnosis of PCa were specially recorded. The total number of biopsies during follow-up period and the delay between final diagnosis and MP-MRI were recorded, respectively.

### Statistical Analysis

Statistical analyses were performed using SPSS version 16.0 (SPSS, Chicago, IL). Two-sided p < 0.05 was significant.

Kolmogorov-Smirnov-test and the Levene *F* test were used to test normality and equality of variances of continuous variables, respectively. Mann-Whitney U tests were used to compare the differences of baseline characteristics between included and excluded subjects.

Weighted kappa coefficients were used to assess interreader agreement. The kappa value was interpreted as an indication of poor agreement when kappa was less than 0.4, as an indication of moderate agreement when kappa was 0.4–0.6, and as an indication of substantial agreement when kappa was greater than 0.6.

To analyze diagnostic efficacy of MP-MRI, a priori we stratified baseline PSA level into four categories (0.01–4.00; 4.01–10.00; >10.00 ng/ml and unknown). Receiver operating characteristics (ROC) curve was used, under results of biopsy within three months. Sensitivity, specificity, negative predictive value (NPV), positive predictive value (PPV) and accuracy were determined for the detection of PCa.

To analyze the prediction ability of MP-MRI in detection of PCa, patients were divided into three groups according to PI-RADS: score 1–2; score 3 and score 4–5. Cumulative detection rate of PCa was derived using Kaplan-Meier method, and differences between curves were analyzed by log-rank test. Death and emigration during follow-up resulted in censoring. A cox proportional hazards model was used to identify independent variables for the detection rate of PCa. Results were presented as hazard ratio (HR) and 95% confidence interval (CI). Proportional of hazard over time for MP-MRI, as a categorical variable, was assessed by plotting–log[-log(detection rate)] versus log(follow-up time). Multivariate models were adjusted for age and baseline PSA level. Age was defined as a continuous variable, while baseline PSA level was defined as a categorical variable (0.01–4.00; 4.01–10.00; >10.00 ng/ml). We had 95.6% complete data on baseline PSA value. Missing values were imputed with a code before multifactorial adjustment.

For patients with negative initial biopsy and final diagnosis of PCa, according to MP-MRI results, they were divided into two groups: score 1–2 and score 3–5. Mann-Whitney U tests were used to analyze the differences of baseline PSA level, total number of biopsies and delay between final diagnosis and MP-MRI between two groups.

## Results

### Patients Characteristics

**Fig 2:**
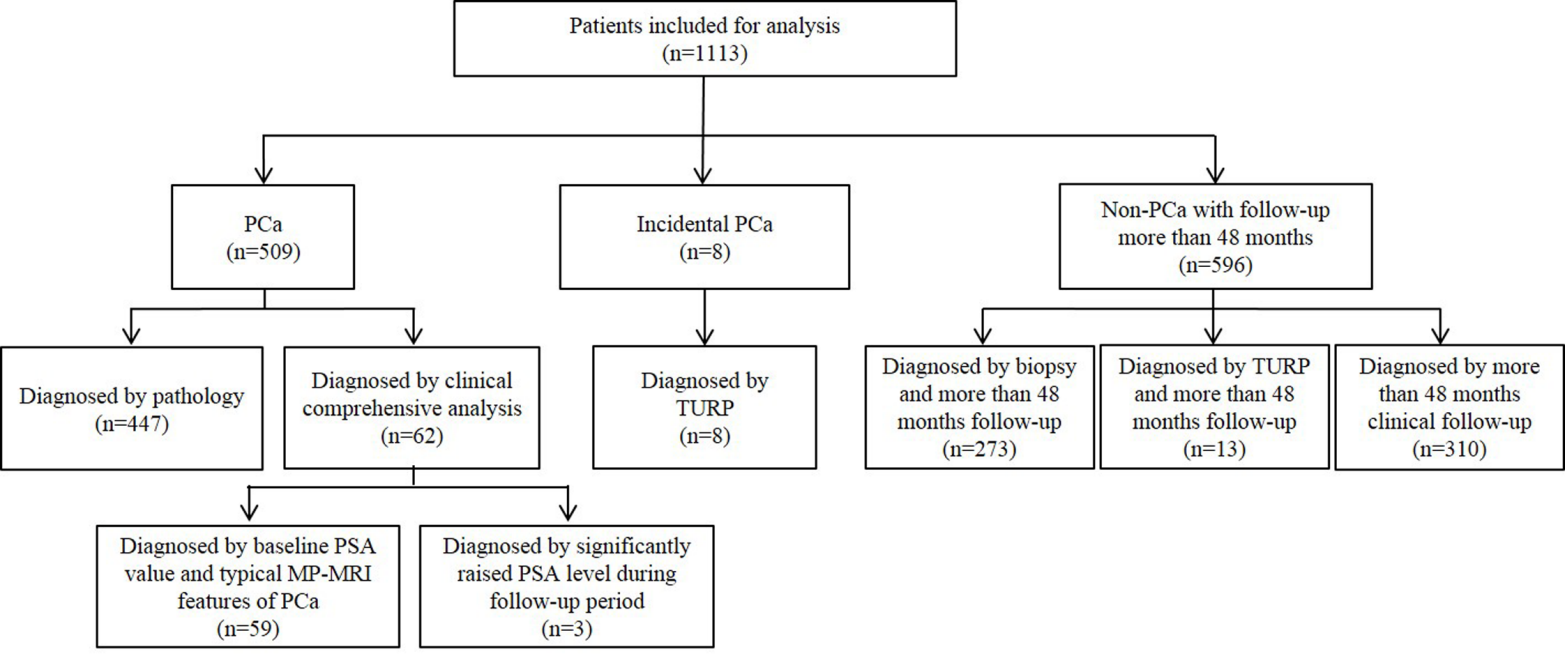
Final Diagnosis and the Diagnosis Evidence of the Cohort. PCa: prostate cancer; TURP: transurethral resection of the prostate; PSA: prostate specific antigen.

A total of 1113 patients (mean age: 70.0 ± 8.3 yr, range: 26–91) were included for data analysis (Fig 2), with median follow-up time of 56.0 months (range: 1–137). There was no significant difference in age between patients included for analysis with those excluded (p = 0.06). The median baseline PSA level was 11.11 ng/ml (range: 0.02–9474.00), which was higher than those excluded (median: 8.87 ng/ml) (p < 0.05). Also, the median score of PI-RADS in patients included was 3, which was greater than those excluded (median: 2) (p < 0.05).

### Interreader Agreement

The degree of agreement was substantial for the overall PI-RADS score between the two readers (kappa = 0.81).

### Diagnostic Efficacy of MP-MRI with PI-RADS in Detection of PCa

Of 1113 patients, 586 patients had biopsy within three months after MP-MRI. A summary of characteristics of patients and biopsy results are provided in Table 2. Detail MP-MRI results are shown in Table 3.

**Table 2:** Baseline Characteristics and Biopsy Results Categorized in Different Baseline PSA Levels.

Variable	PSA 0.01–4.00 ng/ml	PSA 4.01–10.00 ng/ml	PSA >10.00 ng/ml	PSA unknown	Whole
No. of patients	132	345	587	49	1113
Age (yr), mean ± SD (range)	66.8±8.3 (37–86)	68.6±8.5 (26–91)	71.4±7.8 (37–89)	71.4±8.7 (42–84)	70.0±8.3 (26–91)
No. of patients biopsied within three months (rate)	31 (23.5%)	142 (41.2%)	409 (69.7%)	4 (8.2%)	586 (52.7%)
No. of patients biopsied positive within three months (rate)	6 (6/31, 19.4%)	55 (55/142, 38.7%)	278 (278/409, 68.0%)	1 (1/4, 25.0%)	340 (340/586, 58.0%)

PSA: prostate specific antigen.

**Table 3:** Scores of MP-MRI with PI-RADS Categorized in Different PSA Levels.

Score of PI-RADS	PSA 0.01–4.00 ng/ml			PSA 4.01–10.00 ng/ml			PSA >10.00 ng/ml			PSA unknown			Whole		
	Total	Biop-sied*	PCa**	Total	Biop-sied*	PCa**	Total	Biop-sied*	PCa**	Total	Biop-sied*	PCa**	Total	Biop-sied*	PCa**
1	66	10	0	167	54	3	139	65	2	34	2	0	406	131	5
2	36	5	0	58	18	2	35	19	1	6	0	0	135	42	3
3	16	8	2	36	16	7	53	33	17	4	1	0	109	58	26
4	10	4	1	57	31	21	110	88	64	1	0	0	178	123	86
5	4	4	3	27	23	22	250	204	194	4	1	1	285	232	220
Total	132	31	6	345	142	55	587	409	278	49	4	1	1113	586	340

MP-MRI: multiparametric magnetic resonance imaging; PI-RADS: Prostate Imaging Reporting and Data System; PSA: prostate specific antigen; PCa: prostate cancer.

* Only patients with biopsy within three months after prostate MP-MRI were included.

** Only patients, who were pathologically diagnosed with PCa by biopsy within three months after prostate MP-MRI, were included.

For 582 patients with baseline PSA level included for analysis, diagnostic efficacy of MP-MRI for the detection of PCa is presented in Table 4. Each groups showed excellent area under the curve (AUC) (0.88–0.91), suggesting obviously clinically relevant predictive characteristics. Also, MP-MRI showed a very high sensitivity (90.9%-100.0%) and excellent NPV (93.1%-100.0%) in each groups.

**Table 4:** Diagnostic Performance of MP-MRI with PI-RADS.

	PSA 0.01–4.00 ng/ml	PSA 4.01–10.00 ng/ml	PSA >10.00 ng/ml
AUC of the ROC curve	0.88 (0.75–1.00)	0.90 (0.84–0.95)	0.91 (0.87–0.94)
Sensitivity, % (95% CI)	100.0 (100.0–100.0)	90.9 (83.3–98.5)	98.9 (97.7–100.0)
Specificity, % (95% CI)	60.0 (40.8–79.2)	77.0 (68.2–85.9)	61.83 (53.5–70.2)
PPV, % (95% CI)	37.5 (13.8–61.2)	71.4 (60.9–82.0)	84.6 (80.7–88.5)
NPV, % (95% CI)	100.0 (100.0–100.0)	93.1 (87.2–98.9)	96.4 (92.5–100.0)
Accuracy, % (95% CI)	67.7 (51.3–84.2)	82.4 (76.1–88.7)	87.0 (83.8–90.3)

MP-MRI: multiparametric magnetic resonance imaging; PI-RADS: Prostate Imaging Reporting and Data System; PSA: prostate specific antigen; AUC: areas under the curve; ROC: receiver operating characteristic; CI: confidence interval; PPV: positive predictive value; NPV: negative predictive value.

### Prediction Role of MP-MRI with PI-RADS in Detection of PCa

**Fig 3:**
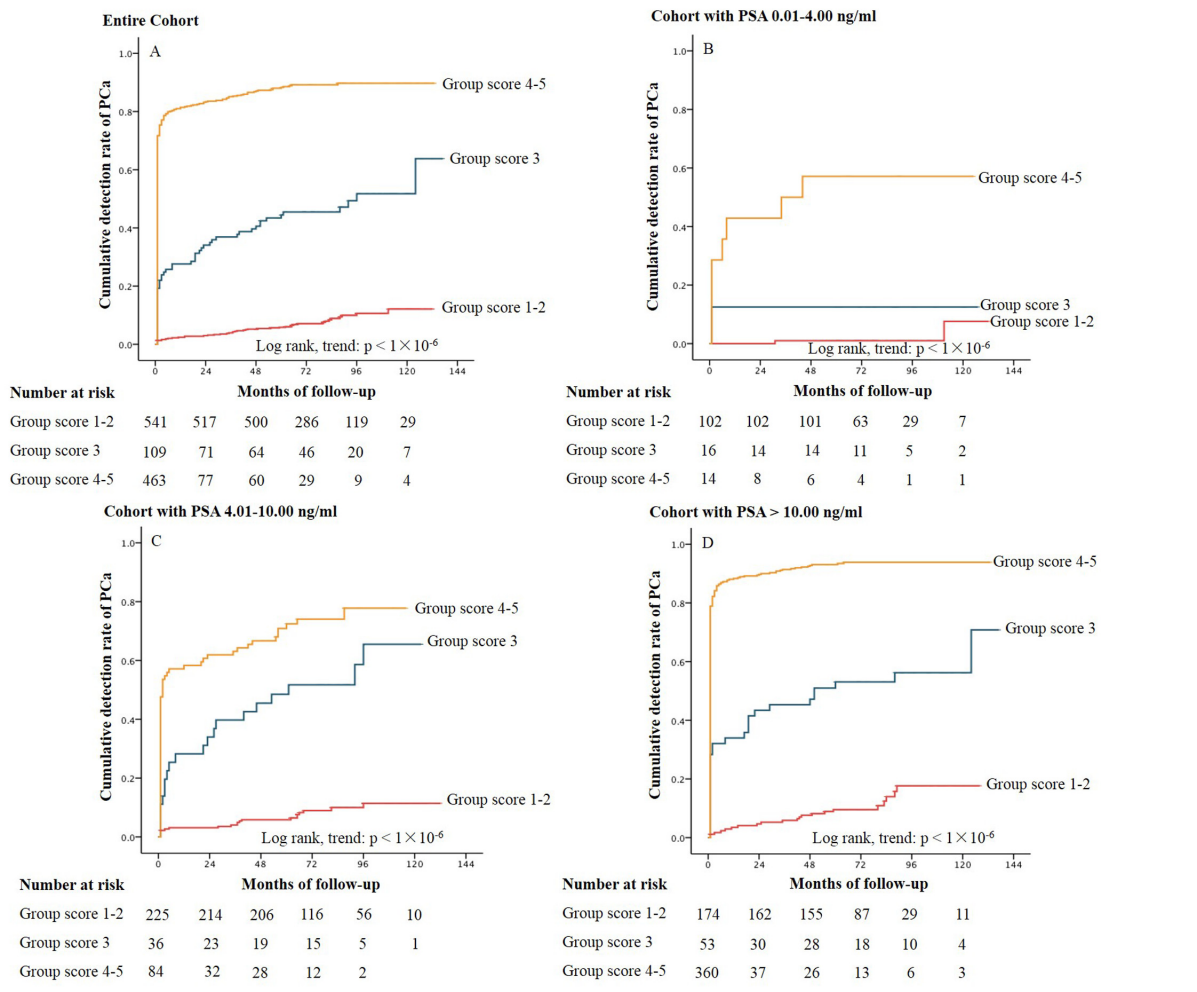
Kaplan-Meier Curves of Cumulative Detection Rate of PCa with MP-MRI in Groups Stratified by PI-RADS for the Entire Cohort (Panel 3A) and the Cohort Categorized in Different Baseline PSA levels (Panel 3B-3D). PCa: prostate cancer; MR-MRI: multiparametric magnetic resonance imaging; PI-RADS: prostate imaging reporting and data system. PSA: prostate specific antigen.

Patients were categorized by scores of PI-RADS: 541 in group score 1–2; 109 in group score 3; 463 in group score 4–5. The detection rate of PCa at the 1-, 5- and 10-year was 2.4%, 5.8% and 12.1% in group score 1–2; 27.6%, 44.4% and 51.7% in group score 3; 81.4%, 88.3% and 89.7% in group score 4–5, respectively (p < 0.001) (Fig 3A). In patients with baseline PSA 0.01–4.00 ng/ml, the corresponding values were 0.0%, 1.0% and 7.6%; 12.5%, 12.5% and 12.5%; 42.9%, 57.1% and 57.1% (Fig 3B). The corresponding values for baseline PSA 4.01–10.00 ng/ml were 3.1%, 5.8% and 11.4%; 28.2%, 48.5% and 65.5%; 58.3%, 72.4% and 77.8% (Fig 3C). The corresponding values for baseline PSA >10.00 ng/ml were 3.5%, 9.6% and 17.7%; 34.0%, 53.1% and 56.2%; 88.3%, 93.1% and 93.9% (Fig 3D). Obviously, the cumulative detection rate of PCa increased obviously as the score of PI-RADS got higher.

Unadjusted and multivariable adjusted hazard ratios with cox proportional hazards model are presented in Table 5. In univariate analyses, older age, greater baseline PSA level and higher score of PI-RADS were all significantly associated with an increased risk of detection rate of PCa. In adjusted analysis with age and baseline PSA, score of MP-MRI with PI-RADS was still the independent risk factor while age not.

**Table 5:** Risk Factors of the Detection Rate of PCa Analyzed by Cox Proportional Hazard Regression Model.

Variable		Unadjusted model		Adjusted model	
	Events, no.	HR (95% CI)	p value	HR (95% CI)	p value
Age (per 1 yr)	509/1113	1.04 (1.03–1.05)	<0.001	1.01 (1.00–1.02)	0.27
Baseline PSA level (ng/ml)					
0.01–4.00 (13%)	12/132	1 (reference)		1 (reference)	
4.01–10.00 (32%)	102/345	3.59 (1.97–6.53)	<0.001	2.54 (1.40–4.63)	0.002
>10.00 (55%)	388/587	10.81 (6.08–19.22)	<0.001	4.42 (2.46–7.94)	<0.001
Score of PI-RADS					
1–2 (49%)	44/541	1 (reference)		1 (reference)	
3 (10%)	53/109	7.24 (4.85–10.80)	<0.001	6.43 (4.29–9.65)	<0.001
4–5 (41%)	412/463	25.09 (18.23–34.54)	<0.001	18.58 (13.36–25.84)	<0.001

PCa: prostate cancer; HR: hazard ratio; CI: confidence interval; PSA: prostate specific antigen; PI-RADS: Prostate Imaging Reporting and Data System.

### Patients with Score 5 of PI-RADS

**Fig 4:**
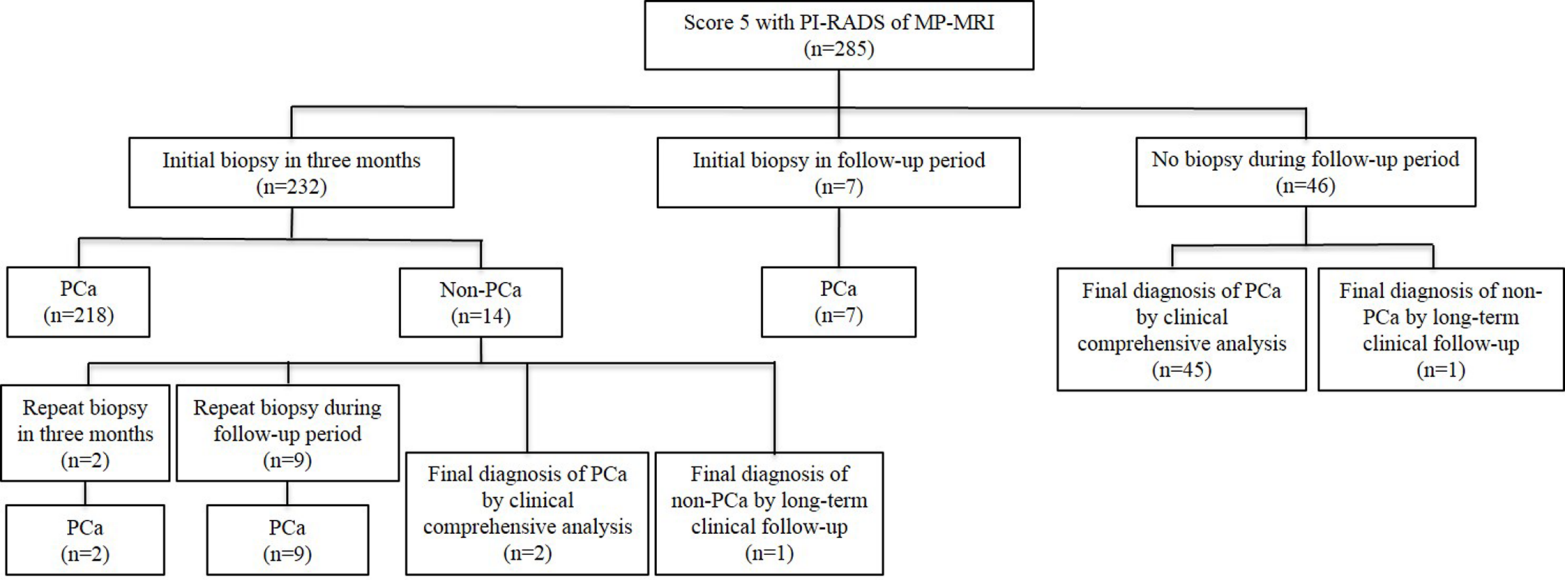
Findings of patients with score 5 of PI-RADS during follow-up period. PI-RADS: Prostate Imaging Reporting and Data System; MR-MRI: multiparametric magnetic resonance imaging; PCa: prostate cancer.

Totally, score 5 of PI-RADS were diagnosed in 285 patients (Fig 4). At the end of follow-up, 283 of 285 patients (99.3%) were diagnosed with PCa. Eleven of 14 patients with negative initial biopsy within three months were pathologically diagnosed with PCa by repeat biopsy and 2 patients were diagnosed with PCa by clinical comprehensive analysis. The rate of PCa detection at TRUS-guided repeat biopsy was 100.0% (11/11).

### Patients with Negative Initial Biopsy and Final Diagnosis of PCa

Of 1113 patients, 51 patients with negative initial biopsy results were finally diagnosed with PCa. Score 1–2 and score 3–5 were 14 (27.5%) and 37 (72.5%), respectively. There was no significant difference in age or baseline PSA level between two groups (p = 0.50). However, the average number of biopsies times of group score 3–5 was significantly less than that of group score 1–2 (p = 0.01). Meanwhile, the median delay between the final diagnosis of PCa and MP-MRI in patients with score 3–5 was 24.0 months, which was shorter than patients with score 1–2 (42.5 months) (p < 0.01).

## Discussion

According to 2013 European Association of Urology guidelines [3], the main tools to diagnose PCa include DRE, PSA, and TRUS-guided biopsy. However, a quantity of problems remain with the current PCa diagnostic pathway [11]. It results in over-diagnosis and over-treatment while missed diagnosis also occurs [12,13]. Avoiding unnecessary biopsies without missing PCa depends largely on the accurate detection of PCa [14].

Recently, several studies reported that advances in MRI made it a promising role in the detection of PCa, with a multiparametric approach and a unified scoring system [15–17]. However, with biopsy results as reference standard, these studies still have the problem of the intrinsic limitation of negative biopsies, as biopsy results can not exclude the risk of PCa [18]. Furthermore, no follow-up data were obtained to evaluate possibly missed biopsies in these studies [18]. Rosenkrantz et al. reported a study with whole-gland pathology as reference standard [19]. However, this study population leads to high concerns regarding applicability, as radical prostatectomy only applies to part of patients with PCa. To eventually realize MP-MRI into the real-life setting as a diagnostic tool, it should be validated in prospective trials and suitable for general population [9].

To our knowledge, our study is the first to evaluate the diagnostic and predictive role of MP-MRI for PCa with a long-term follow-up. Patients with negative pathology results were followed with repeat examinations of DRE, PSA, TRUS and MRI, according to urologists’ choice based on individual patient condition. To increase the reliability of final diagnosis of non-PCa, we excluded patients with the diagnosis of non-PCa based on follow-up less than 48 months, which majorly led to the significant difference in PSA and PI-RADS scores between patients included and excluded.

In our study, excellent NPV (93.1%-100.0%) was presented in each group in our study. The ability to predict with high confidence the absence of PCa (NPV) would control the number of unnecessary biopsy times. Especially for patients with PSA 0.01–4.00 ng/ml, a non-negligible percentage has PCa [20–21], which is difficult to detect early [22]. However, excellent sensitivity (100.0%) in this crowd was achieved with MP-MRI, which would decrease the missed diagnosis rate of PCa. But, the high sensitivity and NPV (100.0%) in this cohort may be caused by the limited sample size, which was one of the limitations in our study. The meta-analysis reported by Hamoen et al. showed a pooled sensitivity of 88% (95% CI, 82–93) and specificity of 45% (95% CI, 27–65) in studies using an overall 5-point PI-RADS scale with a threshold of 3 [18]. Our study showed a higher sensitivity (90.9%-100.0%) and specificity (60.0%-77.0%) in each group, which may associate with the exclusion of patients with non-PCa and follow-up less than 48 months.

Our study showed that even at the 5-year the detection rate of PCa was 1.0%, 5.8% and 9.6% in patients with baseline PSA 0.01–4.00 ng/ml, 4.01–10.00 ng/ml and >10.00 ng/ml, respectively. For patients with score 1–2, the clinicians may consider to choose a more refined diagnostic pathway by taking clinical monitoring with PSA level, DRE, TRUS and MP-MRI as further measurement instead of TRUS-guided biopsy directly. This clinical management could improve the positive rate of biopsy while reducing the biopsy complications.

Higher score of MP-MRI was independently associated with a greater risk of the detection of PCa, which goes along to the findings of previous studies [23–25]. Especially, for patients with score 5 of PI-RADS, 99.3% (283/285) was finally diagnosed with PCa and 100.0% (11/11) of patients with repeat biopsy were pathological diagnosed with PCa. While for patients with negative initial biopsy, our study showed that PCa was more likely to be detected with less number of biopsies and shorter time after prostate MP-MRI in score 1–2 than score 3–5. Therefore, although patients with negative initial biopsy results, the clinicians still should maintain active vigilance against PCa when patients with score 4–5 of PI-RADS, especially with score 5.

A couple of limitations of our study warrant mention. A primary limitation was that our MP-MRI data were obtained over a long period of time (2002–2009). At present, MRI at 3 T with the combination of T2WI, DWI and DCEI is often considered state of the art. Although now at our institution these advanced techniques have already been part of the standard prostate MRI protocol, at the time this study was designed and initiated (2002), these multiparametric imaging had not been proposed and widely introduced into clinical practice. Of note, 1.5 T MR scanner still makes up most of the newly installed MRI units in China. The overall scores of MP-MRI were interpreted based on PI-RADS version 1 in our study. A new version, PI-RADS version 2, was proposed by American College of Radiology (ACR) in December 2014 [26]. PI-RADS version 2 was building and updating upon the foundation of version 1. However, it still needs to be validated by numerous studies in the future. Finally, the analysis was based on the overall prostate as a unit, which is also one of the limitations.

## Conclusion

With the development of MP-MRI and the scoring system (PI-RADS), MP-MRI shows excellent diagnostic efficacy and prediction ability of PCa. Prebiopsy MP-MRI with PI-RADS has potential to be a valuable diagnostic and predictive tool for PCa.

## References

[1] Heijmink SW, Fütterer JJ, Strum SS, Oyen WJ, Frauscher F, Witjes JA, et al State-of-the-art uroradiologic imaging in the diagnosis of prostate cancer. Acta Oncol. 2011;50: 25–38. 10.3109/0284186X.2010.578369 21604938

[2] Kirkham AP, Emberton M, Allen C. How good is MRI at detecting and characterising cancer within the prostate? Eur Urol. 2006;50: 1163–75. 16842903 10.1016/j.eururo.2006.06.025

[3] Heidenreich A, Bastian PJ, Bellmunt J, Bolla M, Joniau S, van der Kwast T, et al EAU guidelines on prostate cancer. part 1: screening, diagnosis, and local treatment with curative intent-update 2013. Eur Urol. 2014;65: 124–37. 10.1016/j.eururo.2013.09.046 24207135

[4] Carter HB, Albertsen PC, Barry MJ, Etzioni R, Freedland SJ, Greene KL, et al Early Detection of Prostate Cancer: AUA Guideline. J Urol. 2013;190: 419–26. 10.1016/j.juro.2013.04.119 23659877 PMC4020420

[5] Hoeks CM, Barentsz JO, Hambrock T, Yakar D, Somford DM, Heijmink SW, et al Prostate cancer: multiparametric MR imaging for detection, localization, and staging. Radiology. 2011;261: 46–66. 10.1148/radiol.11091822 21931141

[6] Portalez D, Rollin G, Leandri P, Elman B, Mouly P, Jonca F, et al Prospective comparison of T2w-MRI and dynamic-contrast-enhanced MRI, 3D-MR spectroscopic imaging or diffusion-weighted MRI in repeat TRUS-guided biopsies. Eur Radiol. 2010;20: 2781–90. 10.1007/s00330-010-1868-6 20680293

[7] Villers A, Lemaitre L, Haffner J, Puech P. Current status of MRI for the diagnosis, staging and prognosis of prostate cancer: implications for focal therapy and active surveillance. Curr Opin Urol. 2009;19: 274–82. 10.1097/MOU.0b013e328329a2ed 19325494

[8] Barentsz JO, Richenberg J, Clements R, Choyke P, Verma S, Villeirs G. ESUR prostate MR guidelines 2012. Eur Radiol. 2012;22: 746–57. 10.1007/s00330-011-2377-y 22322308 PMC3297750

[9] Dickinson L, Ahmed HU, Allen C, Barentsz JO, Carey B, Futterer JJ, et al Magnetic resonance imaging for the detection, localisation, and characterisation of prostate cancer: recommendations from a European consensus meeting. Eur Urol. 2011;59: 477–94. 10.1016/j.eururo.2010.12.009 21195536

[10] Heidenreich A. Consensus criteria for the use of magnetic resonance imaging in the diagnosis and staging of prostate cancer: not ready for routine use. Eur Urol. 2011;59: 495–7. 10.1016/j.eururo.2011.01.013 21256671

[11] Abd-Alazeez M, Kirkham A, Ahmed HU, Arya M, Anastasiadis E, Charman SC, et al Performance of multiparametric MRI in men at risk of prostate cancer before the first biopsy: a paired validating cohort study using template prostate mapping biopsies as the reference standard. Prostate Cancer Prostatic Dis. 2014;17: 40–6. 10.1038/pcan.2013.43 24126797 PMC3954968

[12] Schröder FH, Hugosson J, Roobol MJ, Tammela TL, Ciatto S, Nelen V, et al Screening and prostate-cancer mortality in a randomized European study. N Engl J Med. 2009;360(13): 1320–1328. 10.1056/NEJMoa0810084 19297566

[13] Hugosson J, Carlsson S, Aus G, Bergdahl S, Khatami A, Lodding P, et al Mortality results from the Göteborg randomised population-based prostate-cancer screening trial. Lancet Oncol. 2010;11(8): 725–732. 10.1016/S1470-2045(10)70146-7 20598634 PMC4089887

[14] Tanimoto A, Nakashima J, Kohno H, Shinmoto H, Kuribayashi S. Prostate cancer screening: the clinical value of diffusion-weighted imaging and dynamic MR imaging in combination with T2-weighted imaging. J Magn Reson Imaging. 2007;25: 146–52. 17139633 10.1002/jmri.20793

[15] Komai Y, Numao N, Yoshida S, Matsuoka Y, Nakanishi Y, Ishii C, et al High diagnostic ability of multiparametric magnetic resonance imaging to detect anterior prostate cancer missed by transrectal 12-core biopsy. J Urol. 2013;190: 867–73. 10.1016/j.juro.2013.03.078 23542406

[16] Thompson JE, Moses D, Shnier R, Brenner P, Delprado W, Ponsky L, et al Multiparametric Magnetic resonance imaging guided diagnostic biopsy detects significant prostate cancer and could reduce unnecessary biopsies and over detection: a prospective study. J Urol. 2014;192: 67–74. 10.1016/j.juro.2014.01.014 24518762

[17] Junker D, Schafer G, Edlinger M, Kremser C, Bektic J, Horninger W, et al Evaluation of the PI-RADS scoring system for classifying mpMRI findings in men with suspicion of prostate cancer. Biomed Res Int. 2013;2013: 252939 10.1155/2013/252939 24396825 PMC3876774

[18] Hamoen EH, de Rooij M, Witjes JA, Barentsz JO, Rovers MM. Use of the Prostate Imaging Reporting and Data System (PI-RADS) for Prostate Cancer Detection with Multiparametric Magnetic Resonance Imaging: A Diagnostic Meta-analysis. Eur Urol 2014 (in press). 10.1016/j.eururo.2014.10.033 25466942

[19] Rosenkrantz AB, Kim S, Lim RP, Hindman N, Deng FM, Babb JS, et al Prostate cancer localization using multiparametric MR imaging: comparison of Prostate Imaging Reporting and Data System (PI-RADS) and Likert scales. Radiology. 2013;269: 482–92. 10.1148/radiol.13122233 23788719

[20] Orsted DD, Nordestgaard BG, Jensen GB, Schnohr P, Bojesen SE. Prostate-specific antigen and long-term prediction of prostate cancer incidence and mortality in the general population. Eur Urol. 2012;61: 865–74. 10.1016/j.eururo.2011.11.007 22104593

[21] Lucarelli G, Fanelli M, Larocca AM, Germinario CA, Rutigliano M, Vavallo A, et al Serum sarcosine increases the accuracy of prostate cancer detection in patients with total serum PSA less than 4.0 ng/ml. Prostate. 2012;72: 1611–21. 10.1002/pros.22514 22430630

[22] Miller K, Abrahamsson PA, Akakura K, Debruyne FMJ, Evans CP, Klotz L, et al The continuing role of PSA in the detection and management of prostate cancer. Eur Urol Suppl. 2007;6: 327–33.

[23] Rais-Bahrami S, Siddiqui MM, Turkbey B, Stamatakis L, Logan J, Hoang AN, et al Utility of multiparametric magnetic resonance imaging suspicion levels for detecting prostate cancer. J Urol. 2013;190: 1721–7 10.1016/j.juro.2013.05.052 23727310 PMC6301052

[24] Vargas HA, Akin O, Afaq A, Goldman D, Zheng J, Moskowitz CS, et al Magnetic resonance imaging for predicting prostate biopsy findings in patients considered for active surveillance of clinically low risk prostate cancer. J Urol. 2012;188: 1732 10.1016/j.juro.2012.07.024 23017866 PMC5617124

[25] Fradet V, Kurhanewicz J, Cowan JE, Karl A, Coakley FV, Shinohara K, et al Prostate cancer managed with active surveillance: role of anatomic MR imaging and MR spectroscopic imaging. Radiology. 2010;256: 176–83. 10.1148/radiol.10091147 20505068 PMC2897693

[26] American College of Radiology. MR Prostate Imaging Reporting and Data System version 2.0. Accessed Month YYYY. Available: http://www.acr.org/Quality-Safety/Resources/PIRADS/.

